# Neural stem cells for disease modeling and evaluation of therapeutics for Tay-Sachs disease

**DOI:** 10.1186/s13023-018-0886-3

**Published:** 2018-09-17

**Authors:** Mylinh Vu, Rong Li, Amanda Baskfield, Billy Lu, Atena Farkhondeh, Kirill Gorshkov, Omid Motabar, Jeanette Beers, Guokai Chen, Jizhong Zou, Angela J. Espejo-Mojica, Alexander Rodríguez-López, Carlos J. Alméciga-Díaz, Luis A. Barrera, Xuntian Jiang, Daniel S. Ory, Juan J. Marugan, Wei Zheng

**Affiliations:** 1 National Center for Advancing Translational Sciences, National Institutes of Health, Bethesda, MD USA; 2 Center for Molecular Medicine, National Heart, Lung, and Blood Institute, National Institutes of Health, Bethesda, MD USA; 3 Faculty of Health Sciences, University of Macau, Macau, People’s Republic of China; 4 Institute for the Study of Inborn Errors of Metabolism, Faculty of Sciences, Pontificia Universidad Javeriana, Bogotá, Colombia; 5 Chemistry Department, Faculty of Science, Pontificia Universidad Javeriana, Bogotá, Colombia; 6 Diabetic Cardiovascular Disease Center, Washington University School of Medicine, St. Louis, MO USA

**Keywords:** Tay-Sachs disease, Induced pluripotent stem cells, Neural stem cells, Cyclodextrin, HPβCD, δ-tocopherol, Enzyme replacement therapy, Hexosaminidase A, GM2 gangliosidosis, High throughput screening, Drug discovery

## Abstract

**Background:**

Tay-Sachs disease (TSD) is a rare neurodegenerative disorder caused by autosomal recessive mutations in the *HEXA* gene on chromosome 15 that encodes β-hexosaminidase. Deficiency in HEXA results in accumulation of GM2 ganglioside, a glycosphingolipid, in lysosomes. Currently, there is no effective treatment for TSD.

**Results:**

We generated induced pluripotent stem cells (iPSCs) from two TSD patient dermal fibroblast lines and further differentiated them into neural stem cells (NSCs). The TSD neural stem cells exhibited a disease phenotype of lysosomal lipid accumulation. The Tay-Sachs disease NSCs were then used to evaluate the therapeutic effects of enzyme replacement therapy (ERT) with recombinant human Hex A protein and two small molecular compounds: hydroxypropyl-β-cyclodextrin (HPβCD) and δ-tocopherol. Using this disease model, we observed reduction of lipid accumulation by employing enzyme replacement therapy as well as by the use of HPβCD and δ-tocopherol.

**Conclusion:**

Our results demonstrate that the Tay-Sachs disease NSCs possess the characteristic phenotype to serve as a cell-based disease model for study of the disease pathogenesis and evaluation of drug efficacy. The enzyme replacement therapy with recombinant Hex A protein and two small molecules (cyclodextrin and tocopherol) significantly ameliorated lipid accumulation in the Tay-Sachs disease cell model.

**Electronic supplementary material:**

The online version of this article (10.1186/s13023-018-0886-3) contains supplementary material, which is available to authorized users.

## Background

Tay-Sachs disease (TSD) is one of three lysosomal storage diseases classified as GM2 gangliosidoses, along with Sandhoff disease and the AB variant. TSD and Sandhoff disease are caused by mutations in the *HEXA* and *HEXB* genes, respectively. The AB variant is caused by mutations in the *GM2A* gene encoding for the GM2 activator for β-hexosaminidase A [1]. Both TSD and Sandhoff disease are rare neurodegenerative disorders due to a deficiency in the enzyme β-hexosaminidase, which hydrolyzes GM2 ganglioside into GM3 ganglioside. β-Hexosaminidase is a heterodimer that exists in three isoforms: hexosaminidase A (Hex A), hexosaminidase B (Hex B), and hexosaminidase S (Hex S). Hex A is an α/β heterodimer while Hex B and Hex S consist of two β-subunits and two α-subunits, respectively. In TSD patients, mutations in the *HEXA* gene result in misfolded α-subunits that render Hex A and Hex S non-functional [2]. Deficiency of Hex A activity in TSD causes accumulation of GM2 ganglioside in lysosomes, which ultimately results in progressive neurodegeneration.

There are three forms of TSD: acute infantile, juvenile, and adult. The variations of TSD are characterized by the age of onset and level of remaining Hex A activity in patient cells [3]. Acute infantile TSD is the most common and harmful variant which shows progressive decline in muscle strength and loss of motor skills around six months to three years of age. As the disease progresses, the infant’s brain deteriorates which leads to seizures, blindness, loss of cognitive functions, and ultimately death [4].

Currently, there are no effective treatments for Tay-Sachs disease. The main treatment approach involves managing the symptoms of the disease [4]. Enzyme replacement therapy (ERT) is available for treatment of several lysosomal storage diseases such as Gaucher, Fabry, and Pompe disease [5]. Treatment with recombinant human β-hexosaminidase in both human TSD fibroblasts and mouse TSD models decreased lysosomal GM2 accumulation [6, 7]. However, an earlier study failed to show the beneficial effect of ERT in Tay-Sachs disease patients [8]. Cyclodextrin (HPβCD) and δ-tocopherol have been reported to reduce lipid accumulation and decrease the enlarged lysosomes through increasing lysosomal exocytosis [9]. We have observed the therapeutic effect of HPβCD and δ-tocopherol in the induced pluripotent stem cell (iPSC)-derived neural stem cells (NSCs) in NPC1, NPA, Wolman, and Batten (CLN1 and CLN2) diseases [9–13].

Recent advances in stem cell technology have enabled the generation of disease-specific iPSCs from patient somatic cells. These iPSCs can be differentiated into various types of progenitor cells and mature cells such as neurons, cardiomyocytes, hepatocytes, or retinal pigment epithelial cells for modeling diseases in cell-based assays [14, 15]. Due to the availability of large numbers of NSCs derived from patient iPSCs and their disease phenotypes, they have been used as a cell-based model system for evaluating drug efficacy and drug development [10, 11, 13]. In this study, we report the generation of iPSC lines from two TSD patient dermal fibroblast cells. These TSD iPSC lines were further differentiated into NSCs that exhibited a disease phenotype of lipid accumulation and enlarged lysosomes. The treatment of these patient cells with recombinant human Hex A protein dramatically reduced the lipid accumulation in the TSD cells. δ-Tocopherol and hydroxypropyl-beta-cyclodextrin (HPβCD) also ameliorated the lysosomal lipid accumulation and decreased the enlargement of lysosomes in the TSD NSCs. The results demonstrate that the TSD NSCs differentiated from patient iPSCs are a useful disease model for further study of disease pathophysiology and for use as a cell-based model in drug development.

## Methods

### Materials

Three human dermal fibroblast cell lines were purchased from the Coriell Cell Repositories (Camden, NJ): a wild type female (catalog no. GM05659), a female TSD patient (GM00515), and a male TSD patient (GM00221). See Table 1 for details. DMEM medium (11965092), TrypLE Express (12605010), penicillin-streptomycin (15140122), sodium pyruvate (11360070), Essential 8 medium (A1517001), PSC Neural Induction Medium kit (A1647801), StemPro NSC SFM kit (A1050901), Human neural stem cell immunocytochemistry kit (A24354), CytoTune-iPS 2.0 Sendai Reprogramming Kit (A16517), GlutaMax (35050), Hoechst 33342 (H3570), and Nile Red (N1142) were obtained from Thermo Fisher Scientific. Matrigel hESC-Qualified Matrix (354277) was obtained from Corning. Rock Inhibitor Y-27632 (1254) was obtained from Tocris Bioscience (Ellisville, MO). Hyclone Fetal Bovine Serum (SH30071.03) was purchased from GE Healthcare. δ-Tocopherol obtained from Sigma Aldrich was further purified by HPLC to gain purity greater than 99% while hydroxypropyl-beta-cyclodextrin (HPβCD, E0163) was obtained from Roquette America (IL, USA). Black, clear bottom, tissue-culture treated 96-well plates (655090) were purchased from Greiner Bio-One (Monroe, NC).

**Table 1:** Summary of human cell lines used in the study

Subject	Fibroblast ID (Coriell)	iPSC lines	Gender	Genotype
WT	GM05659	HT268A	Male	Wild Type
Tay-Sachs	GM00221	HT134A, E	Male	Homozygous-c.1278insTATC on exon 11 of *HEXA* gene
Tay-Sachs	GM00515	HT151A, C	Female	Heterozygous-c.1278insTATC and Trp392Ter, both mutations on exon 11 of *HEXA* gene

### Generation and characterization of induced pluripotent stem cells

Fibroblasts were cultured in DMEM medium with 15% fetal bovine serum (FBS), 1X penicillin-streptomycin, and 1X sodium pyruvate. Induced pluripotent stem cell (iPSC) lines were generated from three fibroblast lines using the non-integrating CytoTune-iPS 2.0 Sendai Reprogramming Kit (Table 1). Two iPSC clones were generated from each Tay-Sachs disease cell line. The iPSCs were cultured on Matrigel pre-coated tissue culture plates using Essential 8 medium and passaged using an EDTA-based protocol as previously described [16]. All iPSC lines were cultured beyond passage 10, to ensure the clearance of Sendai viruses and the stability of the iPSC cell lines.

Short tandem repeat (STR) DNA analysis was performed by Johns Hopkins University’s Genetic Resources Core Facility on each set of TSD patient fibroblast cells and NSC lines to confirm their identity as derivatives of patient fibroblast lines GM00515 and GM00221.

G-banded karyotyping analysis of iPSCs was conducted using standard cytogenetic protocol by WiCell Research Institute (Madison, WI). The harvested iPSCs were incubated with ethidium bromide and colcemid in a hypotonic solution before being fixed. Twenty randomly chosen cells in metaphase from each iPSC clone were stained with Leishman’s stain and analyzed by G-banding. The immunofluorescence staining and flow cytometry were also used to characterize iPSCs generated. Immunocytochemistry assay was performed on fixed iPSCs using the pluripotent cell markers of Sox2, Oct4, Nanog, Tra-1-60, and SSEA4. Cells were imaged using an INCell 2200 imaging system (GE Healthcare) using 20X objective lens and Cy5, FITC, and DAPI filter sets. For the FACS analysis, iPSCs were also harvested and fixed using 4% paraformaldehyde and washed, followed by a 10 min incubation with 0.2% Tween-20 to permeabilize cell membrane. The cells were then stained with anti-Tra-1-60-FITC and anti-Nanog-AlexaFluor 488. FACS was performed on a BD Accuri C6 Flow Cytometry System.

### Neural stem cell differentiation and characterization

Tay-Sachs disease iPSCs were differentiated into neural stem cells (NSCs) using the PSC neural Induction Medium kit (Thermo Fisher Scientific) according to the manufacture’s protocol. Once iPSCs reached confluence of 70 to 80%, cells were dislodged from plates using 0.5 mM EDTA buffer and seeded onto Matrigel coated 6-well plates at 3 × 10^5^ cells/well in E8 medium with 10 μM of Rock Inhibitor Y-27632. Cells were incubated overnight at 37 °C to attach onto the plate. The culture media was then changed to PSC Neural Induction Medium containing 1X Neural Induction Supplement in Neurobasal Medium (Thermo Fisher Scientific). Media was changed daily for 7 days. On the 7th day of neural induction, the NSCs were dissociated from the plate using Accutase enzyme cell detachment medium (Thermo Fisher Scientific) and seeded into Matrigel pre-coated T75 flasks for further expansion in the Neural Expansion Medium containing 1X Neural Induction Supplement in equal volume of Neurobasal Medium and Advanced DMEM/F-12 (Thermo Fisher Scientific).

A Human Neural Stem Cell ICC kit (Thermo Fisher Scientific) was used to characterize NSCs generated from TSD patients and WT control iPSCs. The anti-PAX6 antibody was replaced with Oct4 to verify that the NSCs were no longer iPSCs. The manufacture’s protocol was followed for the immunofluorescence staining. Cells were then imaged in the INCell 2200 imaging system (GE Healthcare) using a 20X objective lens with Cy5, FITC, and DAPI filter sets. Additionally, Tay-Sachs disease NSCs were sent to the Johns Hopkins University Genetic Resources Core Facility for STR DNA profiling with the FAF’s PowerPlex 16D (Promega) Standard Service Plus Profile Search.

### Neuron differentiation and characterization

Tay-Sachs patient NSCs were further differentiated into neurons using STEMdiff™ Neuron Differentiation Kit and STEMdiff™ Neuron Maturation Kit (StemCell Technologies) according to the manufacture’s protocol. After 7 days of differentiation of Tay-Sachs NSCs to neuronal precursors in 6- well plate using STEMdiff™ Neuron Differentiation medium, cells were dislodged and seeded into Poly-L-ornithine (PLO)/laminin coated black, clear bottom, tissue-culture treated 96-well plate in STEMdiff™ Neuron Maturation medium for another 7–14 days before downstream application. Immunofluorescence staining of several neuronal markers MAP2 (Cell signaling), beta-III-tubulin (Cell signaling), Neurofilament-L (Cell signaling) as well as Nestin (neural stem cell protein marker, BD Bioscience) were performed to characterize the neuronal cells derived from TSD patient and WT control NSCs.

### Nile red assay

Nile Red dye stains the accumulated lipids and lipid droplets in cells. It has been reported that the yellow-gold fluorescence detects cytoplasmic lipid droplets better than red fluorescence [11, 12, 17]. In this experiment, the Nile Red dye staining was used to evaluate the accumulation of lipids in TSD patient NSCs, as well as neuronal cells. Briefly, cells were seeded onto 96-well plates using the NSC maintenance media from the StemPro NSC SFM kit with 5 μM Rock Inhibitor. Plates with TSD NSCs were incubated overnight at 37 °C and Rock Inhibitor was removed the following day. The assay media was replaced and 10% FBS (Hyclone) was added to the medium followed by overnight incubation at 37 °C to load the lipids into the NSC cells. Assay media was then replaced to remove the FBS, and plates were incubated again overnight at 37 °C for the Nile Red assay to be performed the following day. Nile Red powder was reconstituted in DMSO to 1 mM and stored in the dark at − 20 °C until used. In the Nile Red staining assay, the stock solution was diluted to 1 μM in warmed assay media and incubated with cells for 10 mins in the dark at 37 °C. The cells were then washed twice using Dulbecco’s phosphate-buffered saline (DPBS) and fixed in 4% paraformaldehyde for 30 min at room temperature. Hoechst was also added to the fixing step using a 1:5000 dilution. After washing twice with DPBS, 200 μl/well DPBS was added before analyzing in the INCell 2200 imaging system using a 20X objective lens with TR/TR, FITC/YFP, and DAPI/DAPI filter sets.

### LysoTracker Red dye staining

TSD NSCs were seeded and treated with FBS in the same way as described above. For the LysoTracker dye staining, the NSCs were treated with 50 nM LysoTracker Red DND-99 dye (L-7528, Thermo Fisher Scietific) in assay media at 37 °C for 1 h followed by plate washing twice with DPBS. The plates were fixed and stained with Hoechst simultaneously in 4% paraformaldehyde solution with Hoechst dye at a 1:5000 dilution for 30 min at room temperature. Plates were washed twice using DPBS and stored with 100 μl/well DPBS at 4 °C until imaging. Images were acquired using the INCell 2200 imaging system using a 20X objective lens with Texas Red and DAPI filter sets. 

### Filipin staining

Filipin dye was used to stain unesterified cholesterol in cells [18]. Cells were seeded at 2000 cells/well in 100 μl of media in black, clear bottom, tissue culture-treated 96-well plates and cultured for 24 h. The test compounds were dissolved in media and then added at 100 μl /well; the cells were returned to incubation for 4 days. The cells were washed twice with DPBS and fixed with 100 μl/well of 4% paraformaldehyde (PFA) solution at room temperature for 30 min. After washing twice with DPBS, the cells were stained with 50 ng/mL Filipin solution (freshly dissolved in DMSO at 10 mg/mL and then diluted in DPBS) at room temperature for 1 h. The plates were stored in 100 μl/well DPBS at 4 °C after washing two times with DPBS before imaging analysis. On the day of the imaging, cell nuclei were stained with 100 μl/well of 4 μM ethidium homodimer (EthD-1) (Thermo Fisher Scientific) in DPBS at room temperature for 30 min. The plates were imaged using the INCell 2200 imaging system with a 20X or 40X objective lens. A DAPI filter set (excitation = 350 ± 50 nm, and emission = 455 ± 50 nm) and Cy3 filter set (excitation = 543 ± 22 nm, and emission = 604 ± 64 nm) were used to visualize Filipin and EthD-1 staining, respectively.

### GM2 ganglioside immunofluorescence staining

For immunofluorescence staining of GM2, same FBS treatment protocol was used as for the Nile Red staining experiment prior to the staining. Briefly, 10% FBS was added to the assay medium followed by overnight incubation to load the lipids into the cells. Assay media was then replaced to remove the FBS, and plates were incubated again overnight at 37 °C for the GM2 immunofluorescence staining to be performed the following day. Cells were fixed in 4% paraformaldehyde for 30 min, rinsed with PBS, and permeabilized with 0.3% Triton X-100 for 15 min at 4 °C. Cells were incubated with normal goat serum 10% blocking buffer (Life Technologies) and incubated with mouse anti-GM2 antibody (Amsbio, clone MK1–16) overnight at 4 °C. After washing with PBS, a corresponding secondary antibody conjugated with Alexa Fluor 594 was added. Cells were then stained with Hoechst 33342 for 20 min and imaged using an INCell Analyzer 2200 imaging system (GE Healthcare) with 20X objective lens and Texas Red and DAPI filter sets.

### LC-MS/MS analysis of GM2 in TSD NSCs

We further utilized the LC-tandem mass spectrometry (LC-MS/MS) analysis to determine the GM2 content in TSD patient cells following the protocol established previously [19]. Briefly, TSD patients and WT control neural stem cell pellets were collected and suspended in 50 μL of water. The ganglioside GM2 was extracted from cell suspension with protein precipitation with methanol in the presence of internal standard (d3-GM2 (18:0)). The ganglioside GM2 was separated by column-switching high-performance liquid chromatography (HPLC) and monitored by multiple-reaction monitoring (MRM) detection on an Applied Biosystems Sciex 6500QTRAP+ tandem mass spectrometer (MS/MS) equipped with an electrospray ion source. A quality control (QC) sample was prepared from pooled study samples and injected every 3 study samples to monitor the LC-MS/MS assay performance. All the ganglioside GM2 species in QC injections demonstrate CV < 15%. The protein precipitate from extraction was dissolved in a mixture of 2% CHAPS (100 μL) and 1% SDS (750 μL), and the protein was measured with BCA assay. The relative quantification data were provided as area ratios of GM2 species to internal standard, which were normalized to the protein.

### Recombinant β-hexosaminidases production in Pichia pastoris GS115

Recombinant Hex A was produced in the methylotrophic yeast *Pichia pastoris* GS115 as previously described [20]. Briefly, cDNA of α- (GenBank AAH84537) and β- (GenBank AAH17378) subunits of human β-hexosaminidases were codon-optimized for *P. pastoris* and inserted into a pPICK9k vector. Constructs pPIC9k-alpha or pPIC9k-beta were co-transformed into *P. pastoris* GS115. Clones were evaluated at a shake flask scale, and that with highest activity was used for production of recombinant human Hex A (rhHexA). Cultures were prepared in a modified FM22 saline media and protein production conditions were performed as previously described [20, 21].

Recombinant protein was purified from culture media thanks to the presence of the α-factor secretion signal, and purified by ion exchange chromatography as previously described [20]. β-Hexosaminidase activity was assayed by using 4-methylumbelliferyl-β-D-acetyl-glucosaminide (MUG, Sigma-Aldrich) or 4-methylumbelliferyl-β-D-acetyl-glucosaminide sulfate (MUGS, Calbiochem) substrates [20, 22]. One unit (U) was defined as the amount of enzyme hydrolyzing 1 nmol of substrate per hour. Specific hexosaminidase activity was expressed as U/mg of total protein determined by a Lowry assay.

### Enzyme replacement therapy

TSD NSCs were seeded onto 96-well plates pre-coated with Matrigel using the NSC maintenance media formulated from the StemPro NSC SFM kit containing 5 μM Rock Inhibitor. Plates were incubated at 37 °C overnight and the medium was changed the following day to remove Rock Inhibitor; 100 nM of Hex A recombinant enzyme was incubated with TSD NSCs for 4 h at 37 °C in NSC maintenance media. The media was changed to remove excess recombinant enzymes not taken up by the cells and other components in the buffer of enzyme stock that are not suitable for neural stem cell culture. Because the neural stem cell culture medium does not containing serum and regular lipid components, 10% FBS was added to the medium to facilitate lysosomal storage of lipids. Therefore, we added the enzyme first and wash the cells 4 h later to replace with the medium containing 10% FBS for cell culture overnight. NSCs were cultured in NSC maintenance medium with or without 10% FBS for 24 h at 37 °C. The Nile Red dye staining was performed on the following day.

### Treatments with HPβCD and δ-tocopherol

On Day 0, TSD NSCs and wild type NSCs were seeded into 96-well plates pre-coated with Matrigel in 100 μl/well of NSC maintenance media from the StemPro NSC SFM kit along with 5 μM Rock Inhibitor. The plates were incubated overnight at 37 °C. The media was changed the next day (Day 1) to remove Rock Inhibitor. The cells were treated with HPβCD and δ-tocopherol at 37 °C for 24 h. On Day 2, the medium was replaced with NSC maintenance medium containing 10% FBS and HPβCD or δ-tocopherol followed by incubation at 37 °C for another 24 h. HPβCD was dissolved in water while δ-tocopherol was dissolved in DMSO to a 100 mM stock. The Nile Red dye staining was performed on Day 3 of the experiment.

### ATP content assay for cell viability

An ATP content assay kit (ATPLite, PerkinElmer) was used to measure cell viability to monitor compound cytotoxicity. Cells were seeded at 2500 cells/well in 100 μl medium in white, solid 96-well plates and incubated for 24 h. Cells were cultured and treated as described above. After 3 days of incubation, 100 μl/well of ATP content reagent mixture (prepared according to the manufacturer’s instruction) was added to the assay plates followed by incubation at room temperature for 2–5 min. The luminescence signal was determined in the luminescence mode of the ViewLux Plate reader (PerkinElmer).

### Data analysis and statistics

Image analysis of Nile Red staining, LysoTracker Red staining, and Filipin staining was performed using INCell Analyzer software (GE Healthcare, version 3.7.2). The Multi-Target Analysis protocol was used for quantification of all three assays. Concentration-response curves were analyzed and IC_50_ values calculated using the Prism software (GraphPad, Inc., San Diego). Results in the figures were expressed as mean of replicates ± standard error of the mean (SEM). Unless otherwise stated, an unpaired t-test was used to test for significance, with * *p* < 0.05, ** *p* < 0.01, and *** *p* < 0.001.

## Results

### Generation and characterization of Tay-Sachs iPSCs

**Fig. 1:**
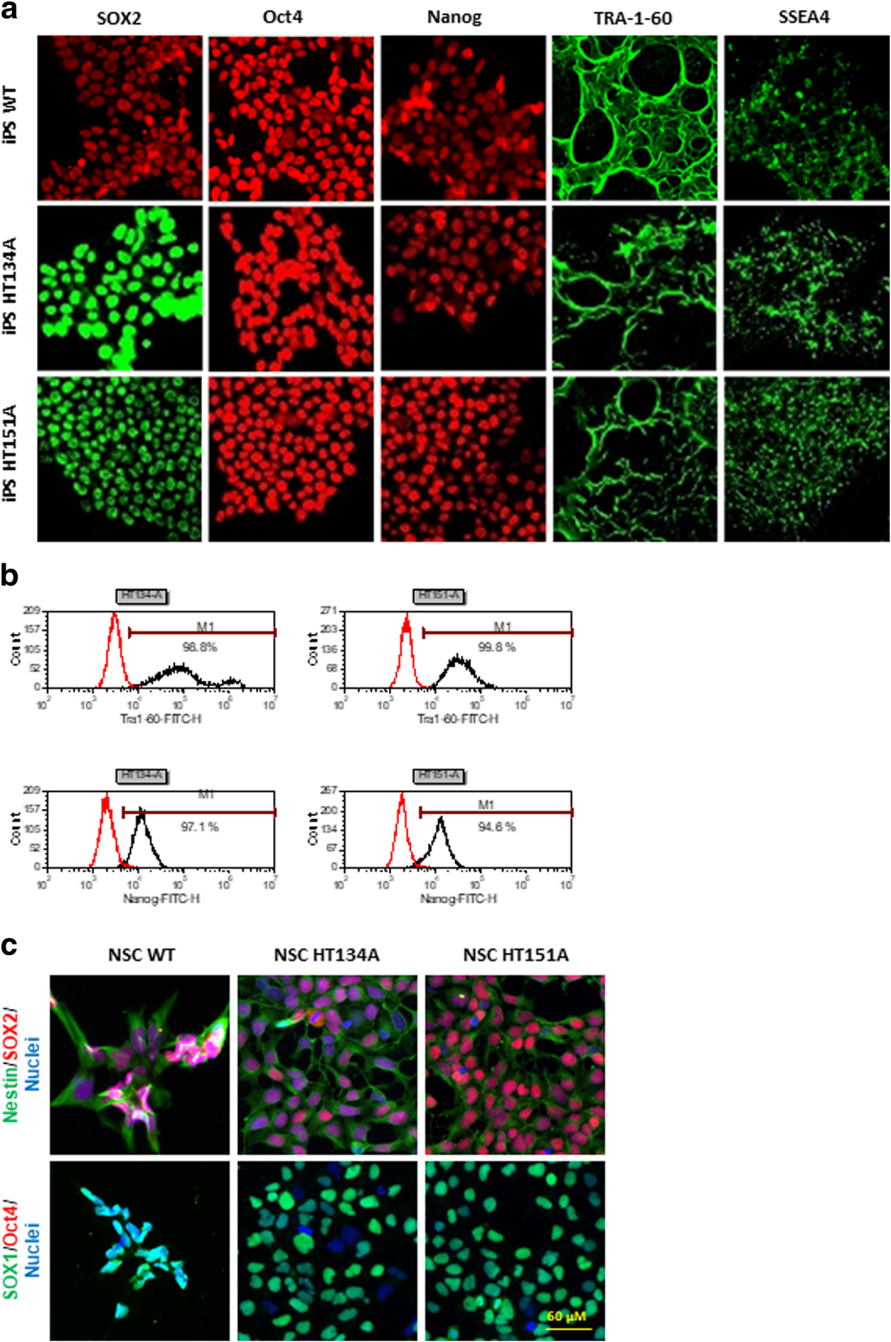
Characterization of Tay-Sachs disease iPSCs and NSCs**. a** The iPSCs derived from TSD patients and WT control fibroblasts expressed pluripotency protein markers SOX2, Oct4, Nanog, TRA-1-60 and SSEA4. **b** Flow cytometry analysis of TSD iPSCs, > 94% of iPSCs expressing Nanog and TRA-1-60 markers. **c** Immunofluorescence staining of TSD and WT NSCs. Nestin, SOX1, and SOX2 are neural stem cell markers while Oct4 is an iPSC marker

Tay-Sachs iPSC lines were generated from two Tay-Sachs patient dermal fibroblasts obtained from Coriell Cell Repositories (GM00221 and GM00515) using the non-integrating Sendai virus reprogramming system that expresses Oct3/4, Sox2, Klf4, and c-Myc factors (Table 1). A patient iPSC line HT134A was established from the fibroblast line GM00221, while another patient iPSC line HT151A was generated from GM00515 (Table 1). No obvious differences in iPSC morphology (Additional file 1: Figure S1A) or growth rate were observed in the TSD iPSC lines compared to the wild type (WT) cells. Flow cytometry analysis showed that the iPSCs expressed the pluripotency markers of TRA-1-60 and Nanog (Fig. 1b, Additional file 1: Figure S1B). Immunofluorescence staining experiments showed positive staining of major pluripotency markers including nuclear markers (SOX2, Oct4, Nanog) and stem cell-specific cell surface markers (TRA-1-60 and SSEA4) (Fig. 1a, Additional file 1: Figure S1A). Furthermore, G-banding karyotyping analysis confirmed the normal karyotype in the TSD iPSC (Additional file 1: Figure S2B). The short tandem repeat (STR) profiling analysis confirmed the cell source of each iPSC line as all 11 checked STR loci matched with its parental Tay-Sachs patient fibroblasts (Additional file 1: Figure S2C). The results demonstrate the successful generation of two TSD iPSC lines.

### Generation and characterization of Tay-Sachs disease neural stem cells

The TSD iPSCs and WT iPSCs were then differentiated into NSCs using the commercial PSC Neural Induction Medium. The NSCs exhibited normal morphology and no significant differences were observed in the differentiation time and cell morphology of patient cells compared to the WT control cells. Immunofluorescence staining of major NSC markers demonstrated that the NSCs differentiated from TSD iPSC lines expressed high level of NSC markers SOX1, SOX2, and Nestin, but not Oct4, a pluripotency marker only expressed in iPSCs (Fig. 1c, Additional file 1: Figure S1C).

### TSD NSCs exhibited lipid accumulation in lysosomes and enlarged lysosomes

**Fig. 2:**
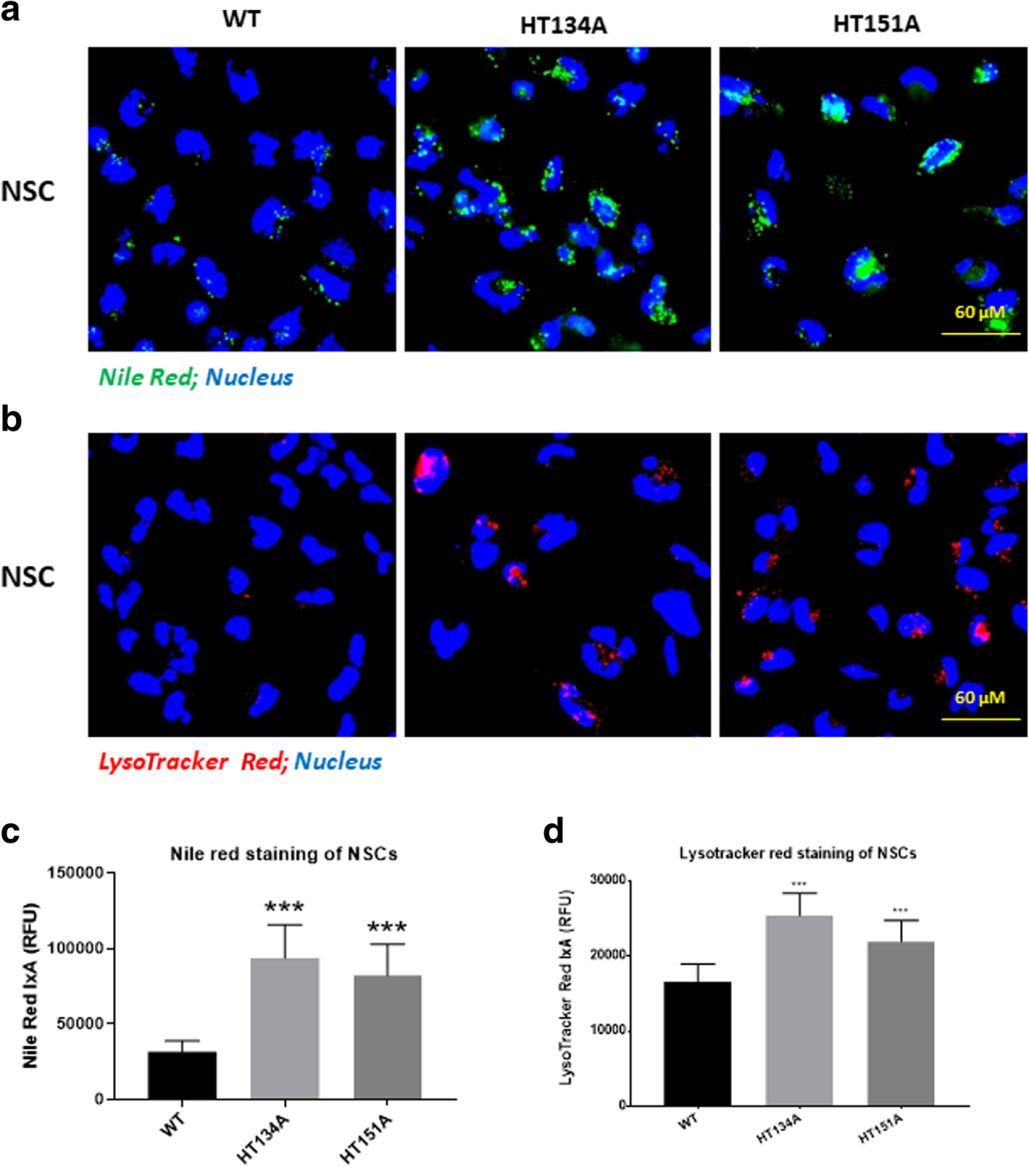
Nile Red and LysoTracker Red staining of TSD NSCs. **a** Images of increased intensity of Nile Red staining in TSD NSC compared to WT NSCs after 24 h addition of 10% FBS. The yellow/gold fluorescence of Nile Red excites and emits at 450-500 nm and 528 nm, respectively. **b** LysoTracker Red staining in TSD NSCs. **c** Intensity of Nile Red staining in TSD NSCs after FBS treatment. **d** Intensity of LysoTracker Red staining in Tay-Sachs disease NSCs after FBS treatment. Data are displayed as mean ± SD. **** *p* < 0.0001 compared to WT control. IxA, integrated cell intensity

To examine the lipid accumulation in lysosomes, a Nile Red dye staining assay was used for visualizing nonpolar lipid in the patient cells [9]. Both TSD NSC lines (HT134A and HT151A) showed increased Nile Red dye staining, indicating accumulation of lipids in lysosomes after loading with lipids in FBS for 24 h (Fig. 2a, Additional file 1: Figure S3A and 3B). In comparison to WT NSCs, the Nile Red staining in NSC HT134A and HT151A lines was two-fold. (Fig. 2c). These results indicated a significant accumulation of nonpolar lipid in both TSD patient iPSC-derived NSCs.

To determine whether the lysosomes were enlarged in those TSD patient cells due to the accumulation of lipid, LysoTracker Red dye staining was also carried out to visualize enlarged lysosomes. A sight increase of lysotracker staining in TSD patient NSCs compared to the NSCs derived from WT control iPSCs. In contrast, the NSCs derived from Niemann-Pick disease, type C1 (NPC1) iPSCs showed much stronger Lysotracker fluorescence signal (Fig. 2b, Additional file 1: Figure S3A and 3C), consistent with our previous findings [9, 13, 23].

To evaluate the potential secondary accumulation of other lipids inside the cells, we further carried out the Filipin staining for unesterified cholesterols that accumulates in the lysosomes of many patient cells with lysosomal storage diseases, especially the Niemann Pick disease type C. The Filipin staining was not significantly different in all four TSD patient iPSC-derived NSCs compared to the WT NSCs (Additional file 1: Figure S5), indicating an absence of lysosomal accumulation of unesterified cholesterol in these TSD patient cells.

Together, these results revealed a disease phenotype of significant increase in lipid accumulation in both TSD NSC lines differentiated from patient iPSCs. Thus, these patient NSCs may serve as a cell-based disease model to study disease pathology to evaluate drug efficacy.

### GM2 ganglioside accumulation in TSD NSCs

To examine the accumulation of GM2 gangliosides in TSD patient cells, immunofluorescence staining of GM2 were carried out. No significant difference was observed in both TSD patient NSCs compared to the WT control (Additional file 1: Figure S4A and 4C).

**Fig. 3:**
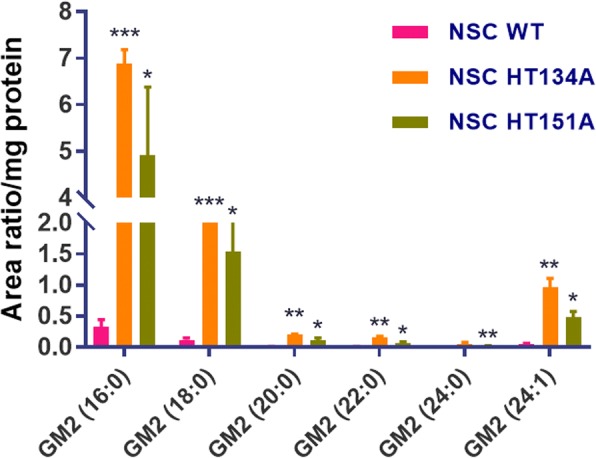
LC-MS/MS analysis of GM2 profile in TSD NSCs. The relative quantification data were provided as area ratios of GM2 species to internal standard, which were normalized to the protein level. Significant elevation of GM2 level was detected in TSN NSCs compared to the WT NSCs. All samples were run in the same batch. Data are displayed as mean ± SD. * *p* < 0.05, ** *p* < 0.01, *** *p* < 0.001, compared to the WT control

GM2 profiling was further performed with LC-MS/MS analysis. In contrast to the immunofluorescence staining data, LC-MS/MS analysis showed significant elevation of GM2 levels in both TSD patient cell lines (NSC HT134A and NSC HT151A) compared to the WT control NSCs (Fig. 3).

### Recombinant human Hex A reduced lipid accumulation in TSD NSCs

**Fig. 4:**
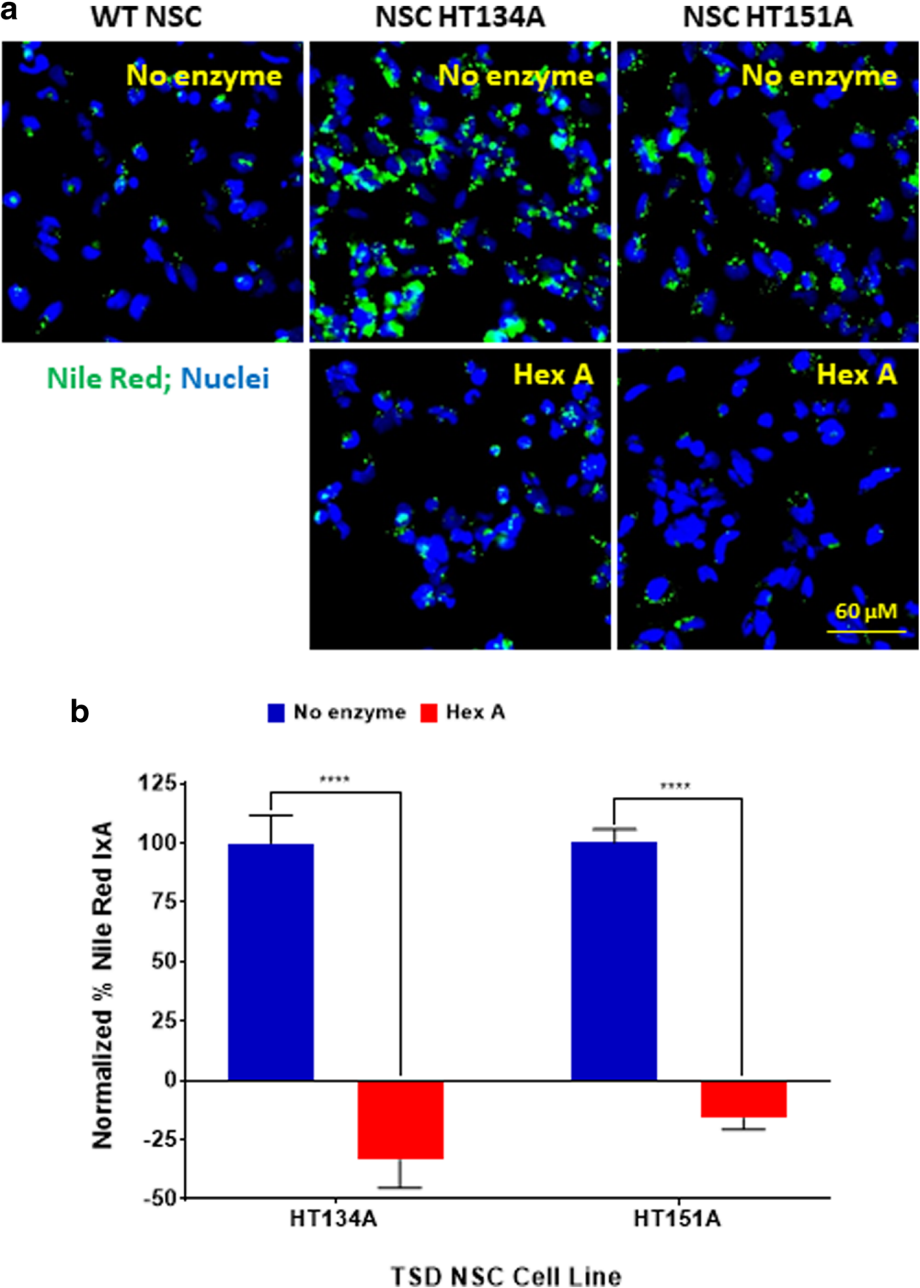
Effect of hexosaminidase A (Hex A) on lipid accumulation in TSD NSCs. **a** Nile Red staining of NSC HT134A and NSC HT151A after 4 h incubation with Hex A. **b** Normalized percent Nile Red IxA of TSD NSCs after treatment with Hex A. Nile Red IxA values of TSD NSCs treated with HexA were normalized to untreated TSD NSCs as 100% and Nile Red IxA of WT NSCs as 0%. Data are mean ± SEM (*n* = 30), **** *p* < 0.0001, compared to each other. IxA, integrated cell intensity

To further validate this cell-based disease model, we evaluated the effect of recombinant human Hex A protein on rescuing the disease phenotype of TSD NSCs. Treatment of NSCs with 100 nM Hex A significantly reduced Nile Red dye staining in TSD NSC lines (HT134A and HT151A) (Fig. 4). Nile Red dye staining in both NSC lines of HT134A and HT151A was reduced to a low level similar to WT cells, indicating significant reduction of lipid accumulation in lysosomes. These results demonstrate that recombinant Hex A treatment rescued the disease phenotype in the TSD NSCs.

### δ-Tocopherol and HPβCD reduced lipid accumulation in TSD NSCs

**Fig. 5:**
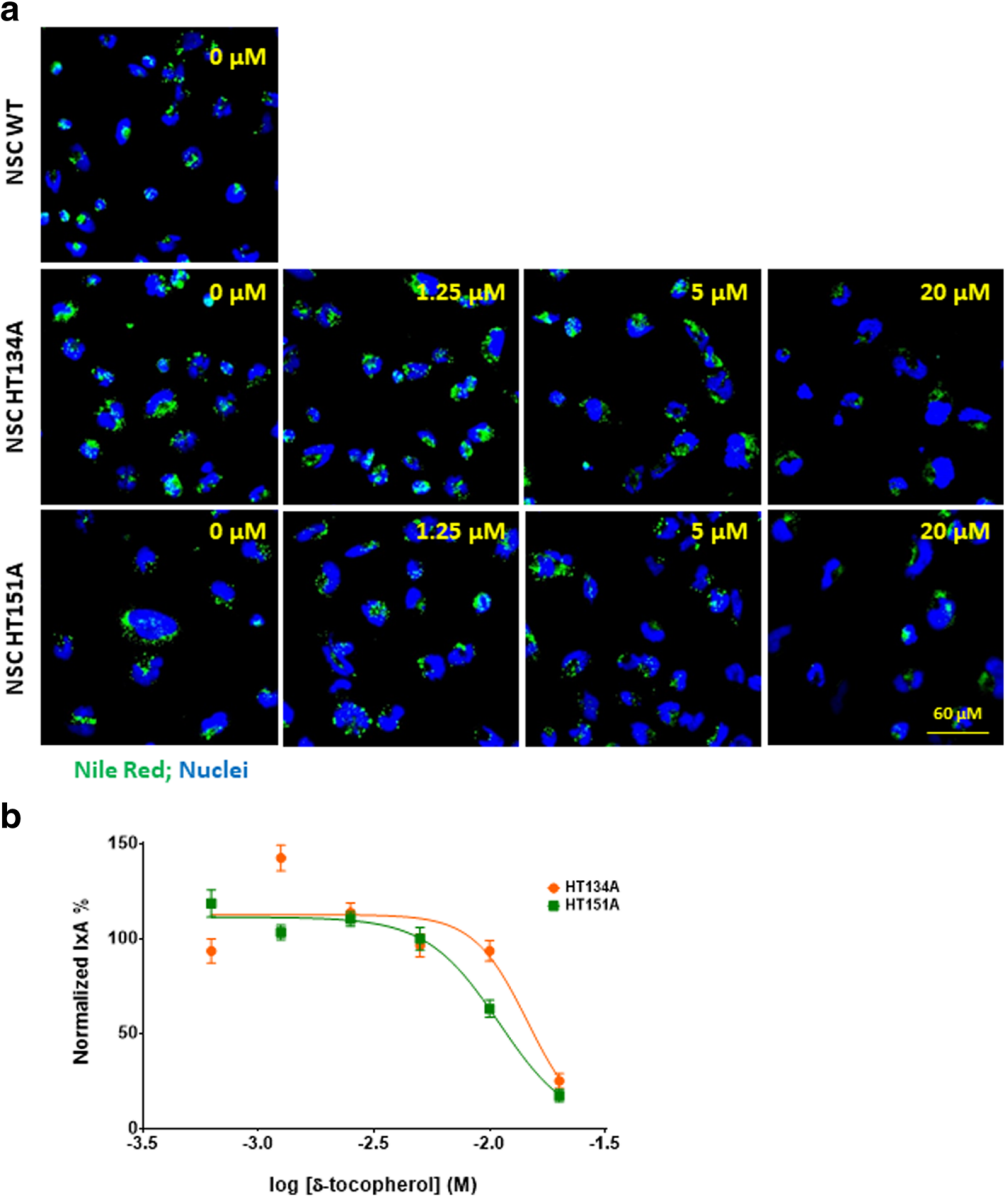
Reduction of lipid accumulation in TSD NSCs after treatment with δ-tocopherol. **a** Nile Red staining of NSC HT134A and HT151A after 48 h of treatment with δ-tocopherol ranging from 0 to 20 uM. **b** Normalized percent IxA of TSD NSCs after treatment with δ-tocopherol (*n* = 40; SEM). Nile Red IxA of TSD NSCs treated with δ-tocopherol were normalized to IxA of untreated TSD NSCs set as 100% and IxA of WT NSCs set as 0%. IxA, integrated cell intensity

**Fig. 6:**
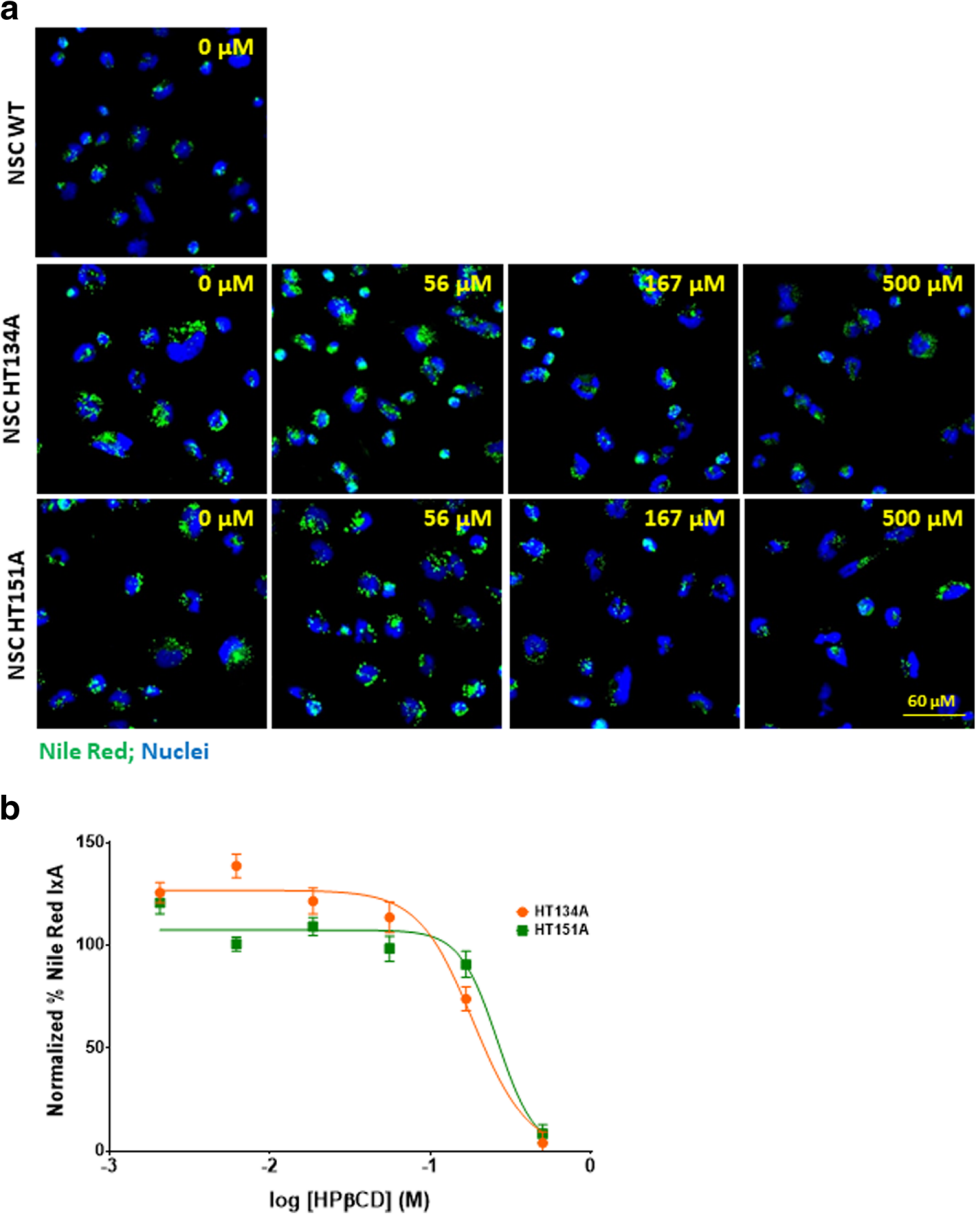
Reduction of lipid accumulation in TSD NSCs after treatment with HPβCD. **a** Nile Red staining of TSD NSC HT134A and HT151A after 48 h treatment with HPβCD ranging from 0 to 500 uM. **b** Normalized percent Nile Red IxA of TSD NSCs after treated with HPβCD (n = 40; SEM). Nile Red IxA of HPβCD treated TSD NSCs were normalized to untreated TSD NSCs IxA as 100% and WT NSCs IxA as 0%. IxA, integrated cell intensity

δ-Tocopherol and HPβCD have been reported to significantly reduce lysosomal accumulation of cholesterol and decrease the enlargement of lysosomes in NPC1 and NPA cells [10, 13, 23]. The positive effects of δ-tocopherol were previously observed in the TSD fibroblasts [9]. Here, we examined the effects of δ-tocopherol and HPβCD on lipids accumulated in TSD NSCs using the Nile Red dye staining. We found that both compounds reduced Nile Red dye staining in a dose-dependent manner (Figs. 5 and 6). The half maximal effective concentration (EC_50_) values of δ-tocopherol were 14.5 μM in HT134A and 10.9 μM in HT151A lines. Treatment with 20 μM δ-tocopherol displayed a 75% and 83% decrease of Nile Red staining compared to the untreated patient cell lines, HT134A and HT151A, respectively (Fig. 5b). Treatment with 500 μM HPβCD dramatically reduced the Nile Red dye staining to a level similar to WT NSCs, with a 97% reduction in the TSD HT134A line and a 92% decrease in the HT151A line. The EC_50_ values for TSD NSCs treated with HPβCD were 183.8 μM in cell line HT134A and 261.1 μM in cell line HT151A (Fig. 6b). Together, the results demonstrate that δ-tocopherol and HPβCD significantly ameliorated the lipid accumulation in the TSD NSCs.

**Fig. 7:**
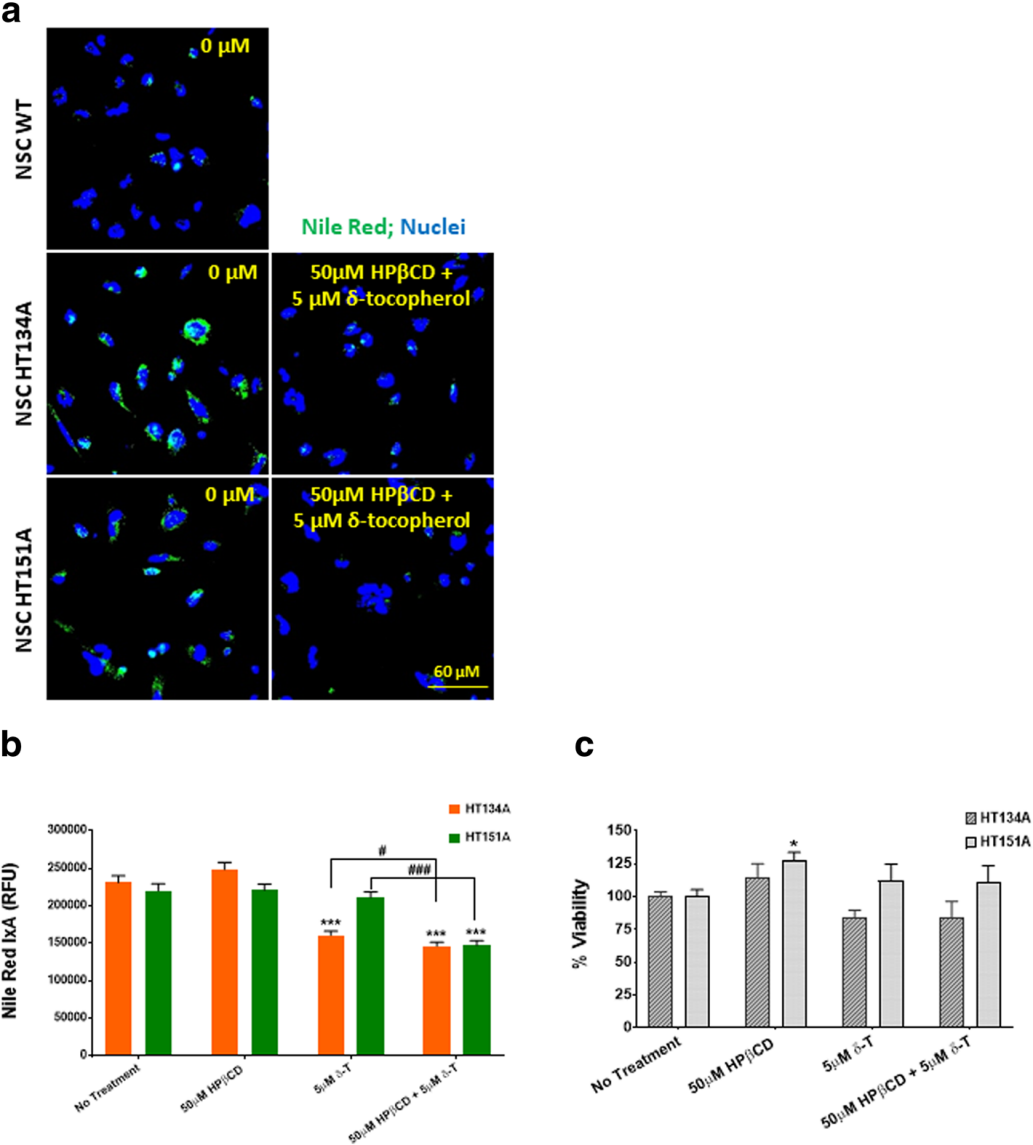
Combination treatment of HPβCD and δ-tocopherol reduces lipid accumulation in TSD NSCs. **a** Nile Red staining images of TSD NSCs compared to WT NSCs following compound combination treatment. **b** Nile Red IxA of NSC HT134A and HT151A after 48 h incubation with HPβCD and δ-tocopherol. **c** Cell viability of TSD NSCs after compound combination treatment. * *p* < 0.5, *** *p* < 0.001 compared to untreated control. # *p* < 0.05, ### *p* < 0.001 compared to each other. IxA, integrated cell intensity

To improve the therapeutic effect and reduce the drug concentrations needed to achieve the maximal response, a combination therapy of both δ-tocopherol and HPβCD was also tested. This combination showed a significantly decrease of lipid accumulation in TSD NSCs (Fig. 7). A combination of 50 μM HPβCD and 5 μM δ-tocopherol, both at lower concentrations than either one alone, was effective in reducing lipid accumulation. At these concentrations (50 μM of HPβCD and 5 μM of δ-tocopherol), no significant reduction of lipid accumulation was observed when either compound was used alone. The combination of 5 μM δ-tocopherol and 50 μM HPβCD significantly decreased lipid accumulation to levels comparable to WT cells, suggesting a synergistic effect (Fig. 7a, b). Additionally, an ATP content assay confirmed the cell viability from different experimental groups (Fig. 7c).

### Neuronal cell differentiation from Tay-Sachs iPS cells

**Fig. 8:**
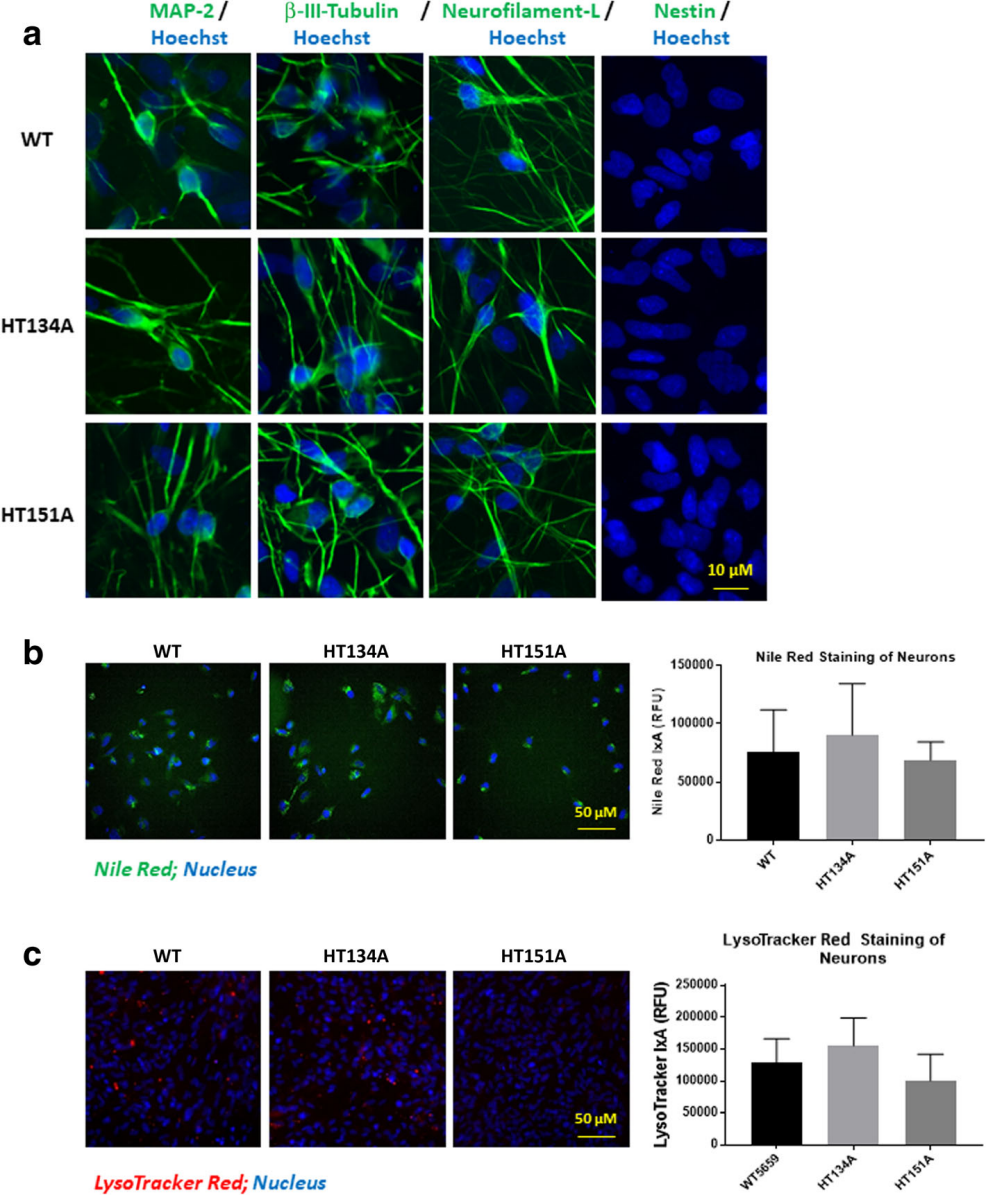
Neuronal cell differentiation from Tay-Sachs iPSCs. **a** Immunofluorescence staining of TSD and WT neurons. MAP2, β-III-tubulin and Neurofilament-L are neuronal cell protein markers while Nestin is a neural stem cell marker, serving as the negative control. **b** Nile red staining on TSD and WT neurons. **c** LysoTracker red staining on TSD and WT neurons. No significant increase of Nile red staining or LysoTracker red staining was observed in TSD neurons compared to the WT control cells. Data are displayed as mean ± SD. * *p* < 0.05, compared to the WT control. IxA, integrated cell intensity

We further differentiated TSD NSCs and WT NSCs into neuronal cells. Immunofluorescence staining of major neuronal cell makers demonstrated that the neuronal cells differentiated from TSD and WT NSCs lines expressed high levels of neuronal cell protein markers MAP2, ß-III-Tubulin, Neurofilament-L, but not Nestin (a protein marker of neural stem cells). (Fig. 8a). The results provide evidence for the generation of TSD neurons from patient NSCs cells.

Nile Red staining and LysoTracker Red staining were carried out to evaluate the lipid accumulation and lysosome enlargement in TSD neurons. However, we only observed a small, but not significant increase of both Nile Red fluorescence signal (Fig. 8b) and lysoTracker Red staining signal (Fig. 8c) in one of the patient cell lines, HT131A, but not in HT151 patient line compared to the WT control neurons. 

## Discussion

δ-Tocopherol and HPβCD had been reported to significantly reduce lysosomal accumulation of cholesterol and decrease the enlargement of lysosomes in NPC1 and NPA cells [10, 13, 23].

Recent advances in iPSC technology have enabled the generation of patient iPSC lines from skin fibroblasts and other cells such as peripheral blood mononuclear cells (PBMCs). These patient-derived iPSC are self-renewable and can be differentiated to many cell types previously hard to obtain including neuronal cells, cardiomyocytes, and hepatocytes [13, 24–28]. Neural stem cells can self-renew and also exhibit the characteristic disease phenotype of several lysosomal diseases including Niemann Pick disease type C (NPC), NPA and Wolman disease [10, 11, 13]. Other groups have also employed patient iPSC-derived NSCs or neurons to study disease pathogenesis and phenotypes such as neuropsychiatric diseases [27]. Those patient-derived cells have also been used as cell-based disease models to evaluate drug efficacy and phenotypic high throughput screening (HTS) to identify new lead compounds for drug discovery [10, 11, 13] [29].

In this study, we have successfully generated two Tay-Sachs disease patient iPSC lines and further differentiated them into NSCs that exhibit a characteristic disease phenotype of lysosomal lipid accumulation. GM2 is the primary lipid accumulated in the Tay-Sachs’ patient cells [2]. However, we found that the immunostaining with anti-GM2 antibodies did not reveal difference between the patient cells and wild type control cells. This might be due to the issue with specificity of commercially available GM2 antibodies that are not sensitive enough for the immunostaining experiment. Further study using MS method confirmed the accumulation of GM2 lipid in the patient cells. However, the GM2 measurement using MS method requires a significant amount of resources and cells without the high-throughput necessary for compound screening.

In addition to GM2 ganglioside buildup, secondary accumulation of other lipids including phospholipids, cerebrosides, sphingomyelin, and cholesterol have been reported [30]. Nile Red is a hydrophobic and metachromatic dye, which yields fluorescence varying from deep red to strong yellow-gold depending on the hydrophobicity of the lipid environment [17]. In this study, we found that the increased Nile Red dye staining in TSD patient NSCs could be rescued by recombinant human Hex A protein. Thus, these patient cells can be used as a cell-based disease model and Nile Red staining offers a valid method for evaluating drug efficacy and compound screening. The significant lipid accumulation was not observed in the patient iPSC-derived neurons in our experiments. It could be due to a lack of neuronal maturation. The images of LysoTracker Red and Nile Red staining in neurons also exhibited high variability in individual cells, indicating that the current method may not be sensitive enough for neuronal detection. Currently, production of large amount of neurons is still a bottleneck for compound screening assays. The reproducibility of iPSC-differentiated neurons from batch to batch can not be easily controlled. Therefore, we have used the NSCs [9–12] as a model system for evaluation of compound efficacy and compound screening because NSCs can be produced in large quantities with good reproducibility.

Previously, ERT for TSD was tested in clinical trials through intraventricular delivery and injections into the spinal canal. However, no reduction of GM2 ganglioside accumulation was detected in patients due to the limited amount of the enzyme that was able to reach neural cells [8]. In this study, we found that patient NSCs treated with Hex A exhibited decreased lipid accumulation (Fig. 4). It has been reported that just 10% of normal activity of Hex A is required for patients to be characterized as WT [31]. Therefore, only a small amount of Hex A activity is needed to ameliorate the disease phenotype.

Even though ERT has proven to be an effective treatment in several other lysosomal storage disorders like Gaucher disease, and Pompe diseases, ERT is not feasible for TSD [5] because recombinant Hex A cannot cross the blood-brain barrier (BBB) and therefore cannot be effective for neurological symptoms [8]. The recombinant enzyme may be directly administrated into brain via intrathecal or intraparenchymal injection. But the procedure is invasive and usually not suggested as the best route of treatment due to its high safety risks and costs [32, 33]. Furthermore, ERT for lysosomal storage diseases generally requires frequent administration [34].

Nonetheless, ERT should be further investigated and developed for patients with TSD to ameliorate symptoms in the peripheral body such as muscle weakness, spasm, and paralysis. New technologies and methods are needed for efficient and safe delivery of recombinant Hex A protein into the brain. One of the methods currently under study is using ultrasound to temporarily increase the permeability of the BBB, as well as decreasing the efflux of transporters to allow more passive diffusion of hydrophobic compounds [35]. Additionally, liposomes and their polymer analog, polymerosomes, have been used in conjugation with ligands or polymer chains that allow for specific drug delivery across the BBB [33, 35] and enable ERT for lysosomal storage diseases with neurological effects.

We also evaluated two small molecules, HPβCD and δ-tocopherol, using the TSD patient NSCs. Both compounds effectively reduced lipid accumulation in TSD NSCs. However, a much higher concentration of HPβCD is needed to reduced lipid accumulation in TSD cells compared to NPC1 NSCs [13]. The concentration of HPβCD can be significantly reduced when it was used in combination with δ-tocopherol. It has been reported that both δ-tocopherol and HPβCD decrease cholesterol accumulation in NPC1 cells by inducing lysosomal exocytosis [9, 36]. Although the exact mechanisms of action for both δ-tocopherol and HPβCD in TSD NSCs are unclear, it is possible that there is a synergistic effect when the two compounds are used as a combination therapy.

## Conclusions

Two lines of iPSCs have been generated from TSD patient fibroblasts, and the NSCs derived from these TSD iPSCs exhibit the disease phenotype of lipid accumulation in lysosomes. ERT with recombinant Hex A protein rescues the disease phenotype in the TSD NSCs. HPβCD and δ-tocopherol also reduce the lysosomal lipid accumulation in patient-derived NSCs. Overall, our data demonstrate that the patient-derived NSCs can be used as a cell-based disease model to study disease pathology and perform compound screening for drug development.

## Additional file

Additional file 1: **Figure S1.** Tay-Sachs disease induced pluripotent stem cells (iPSCs) generation and neuronal stem cells (NSCs) differentiation. **Figure S2.** Characterization of Tay-Sachs disease iPSCs. **Figure S3.** Tay-Sachs disease NSCs express increased lipid accumulation and lysosomal size compared to WT NSCs. **Figure S4.** Immunofluorescence staining of GM2 in TSD patient NSCs and neurons.
