# Supplementary material for: Neural stem cells for disease modeling and evaluation of therapeutics for Tay-Sachs disease
Source: Orphanet J Rare Dis. 2018 Sep 17;13:152. doi: 10.1186/s13023-018-0886-3 (PMC6139903; doi:10.1186/s13023-018-0886-3)
Supplement: Supplementary file 1 — Figure S1. Tay-Sachs disease induced pluripotent stem cells (iPSCs) generation and neuronal stem cells (NSCs) differentiation. Figure S2. Characterization of Tay-Sachs disease iPSCs. Figure S3. Tay-Sachs disease NSCs express increased lipid accumulation and lysosomal size compared to WT NSCs. Figure S4. Immunofluorescence staining of GM2 in TSD patient NSCs and neurons. [file 13023_2018_886_MOESM1_ESM.pdf]

Supporting Information for

Neural Stem Cells for Disease Modeling and Evaluation of Therapeutics for Tay-Sachs disease

Mylinh Vu,[a] Rong Li,[a] Amanda Baskfield,[a] Billy Lu,[a] Atena Farkhondeh,[a] Kirill Gorshkov,[a] Omid Motabar,[a] Jeanette Beers,[b] Guokai Chen,[b, c] Jizhong Zou,[b] Angela J. Espejo-Mojica,[d] Alexander Rodríguez-López,[d, e] Carlos J. Alméciga-Díaz,[d] Luis A. Barrera,[d] Xuntian Jiang,[f] Daniel S. Ory,[f] Juan J. Marugan,[a] and Wei Zheng[a]†

[a]National Center for Advancing Translational Sciences, National Institutes of Health, Bethesda, Maryland, USA; [b]Center for Molecular Medicine, National Heart, Lung, and Blood institute, National Institutes of Health, Bethesda, Maryland, USA; [c]Faculty of Health Sciences, University of Macau, Macau, People's Republic of China; [d]Institute for the Study of Inborn Errors of Metabolism, Faculty of Sciences, Pontificia Universidad Javeriana, Bogotá, Colombia; [e]Chemistry Department, Faculty of Science, Pontificia Universidad Javeriana, Bogotá, Colombia
[f]Diabetic Cardiovascular Disease Center, Washington University School of Medicine, St. Louis, Missouri, USA.

†Corresponding authors:

Wei Zheng, Ph.D.
National Center for Advancing Translational Sciences
National Institutes of Health
9800 Medical Center Drive, MSC: 3375
Bethesda, MD 20892
Email: wzheng@mail.nih.gov
Tel.: (301) 217-5251

Supplemental Fig. 1

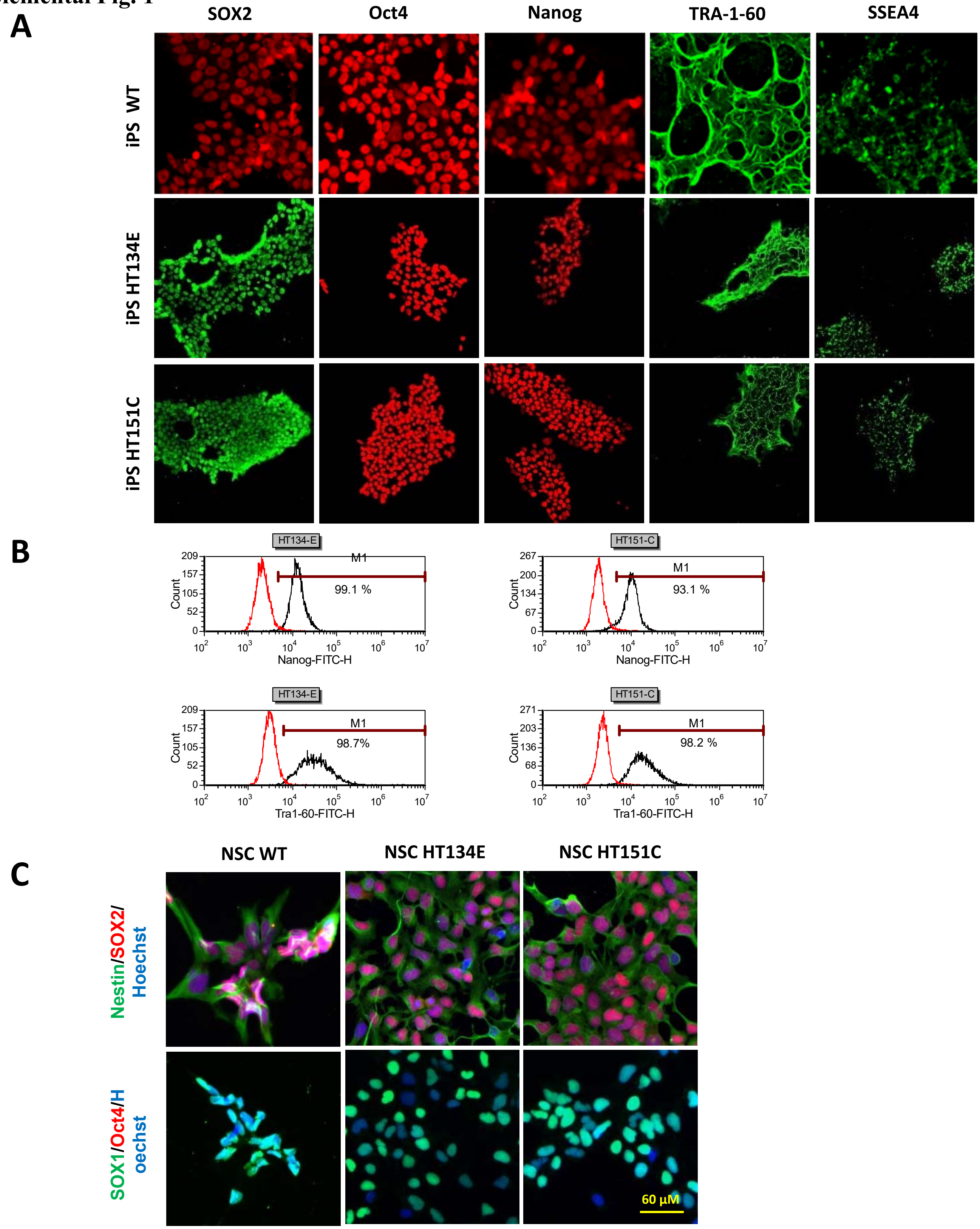

Supplemental Figure 1. Tay-Sachs disease induced pluripotent stem cells (iPSCs) generation and neuronal stem cells (NSCs) differentiation. A) The iPSCs derived from TSD patients and wild type (WT) control fibroblasts expressed pluripotency protein markers SOX2, Oct4, NANOG, TRA-1-60 and SSEA4. B) Flow cytometry analysis of TSD iPSCs shows more than 90% of iPSCs express Nanog and Tra-1-60 markers. C) Immunofluorescence staining of TSD NSCs. Nestin, SOX1, and SOX2 are neural stem cell markers while Oct4 is an iPSC marker.

Supplemental Fig. 2

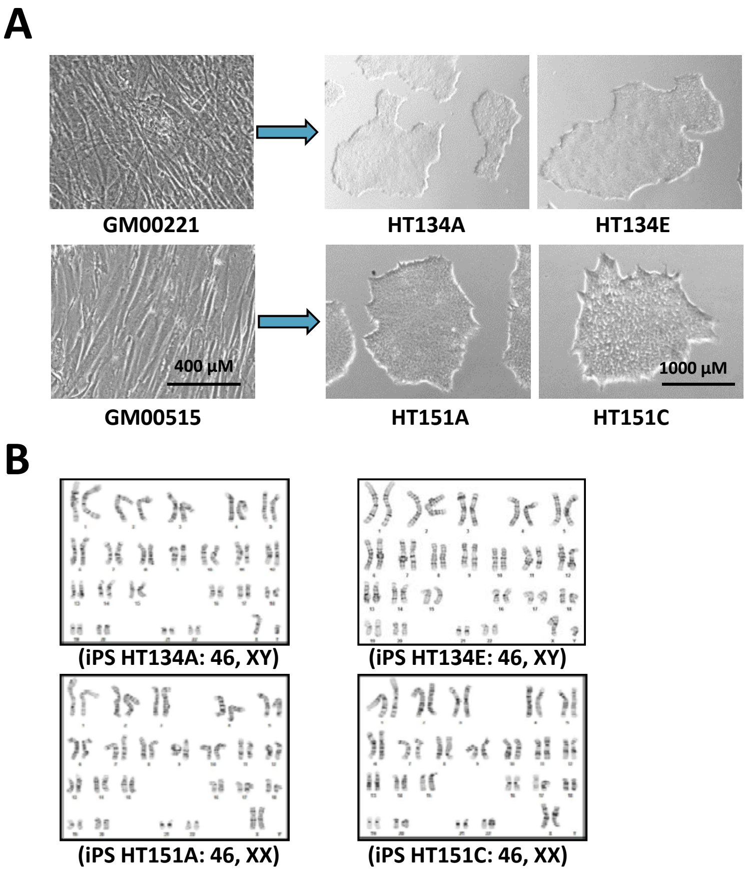

C

Cell line	FIB GM00515	NSC HT151A	NSC HT151C	FIB GM00221	NSC HT134A	NSC HT134E
FGA	19,25	19,25	19,25	20,25	20,25	20,25
TPOX	8,12	8,12	8,12	8,11	8,11	8,11
D8S1179	12,13	12,13	12,13	13,16	13,16	13,16
vWA	14,15	14,15	14,15	14,17	14,17	14,17
Amelogenin	X,X	X,X	X,X	X,Y	X,Y	X,Y
Penta_D	12,12	12,12	12,12	13,15	13,15	13,15
CSF1PO	12,12	12,12	12,12	10,10	10,10	10,10
D16S539	12,14	12,14	12,14	13,13	13,13	13,13
D7S820	11,12	11,12	11,12	8,11	8,11	8,11
D13S317	11,11	11,11	11,11	8,12	8,12	8,12
D5S818	11,12	11,12	11,12	12,13	12,13	12,13
Penta_E	11,13	11,13	11,13	11,14**	11,14	11,14
D18S51	12,17	12,17	12,17	13,15	13,15	13,15
D21S11	29,30	29,30	29,30	31.2,33.2	31.2,33.2	31.2,33.2
TH01	7,9	7,9	7,9	6,10	6,10	6,10
D3S1358	16,17	16,17	16,17	16,18	16,18	16,18
D19S433	N/A	14,14	14,14	N/A	14,14	14,14
D2S1338	N/A	17,25	17,25	N/A	18,24	18,24

** Allelic imbalance

Supplemental Figure 2. Characterization of Tay-Sachs disease induced pluripotent stem cells. A) Phase contrast image of Tay-Sachs disease fibroblast lines, GM00221 and GM00515, after reprogrammed into iPSC HT134A/HT134E and HT151A/HT151C, respectively. B) Normal karyotype of Tay-Sachs disease iPSC. All cell lines displayed normal karyotype. iPS HT134A and iPS HT134E are 46XY while iPS HT151A and iPS HT151C are 46XX. C) STR DNA profiling of Tay-Sachs disease fibroblasts and derived NSC cell lines.

Supplemental Fig. 3

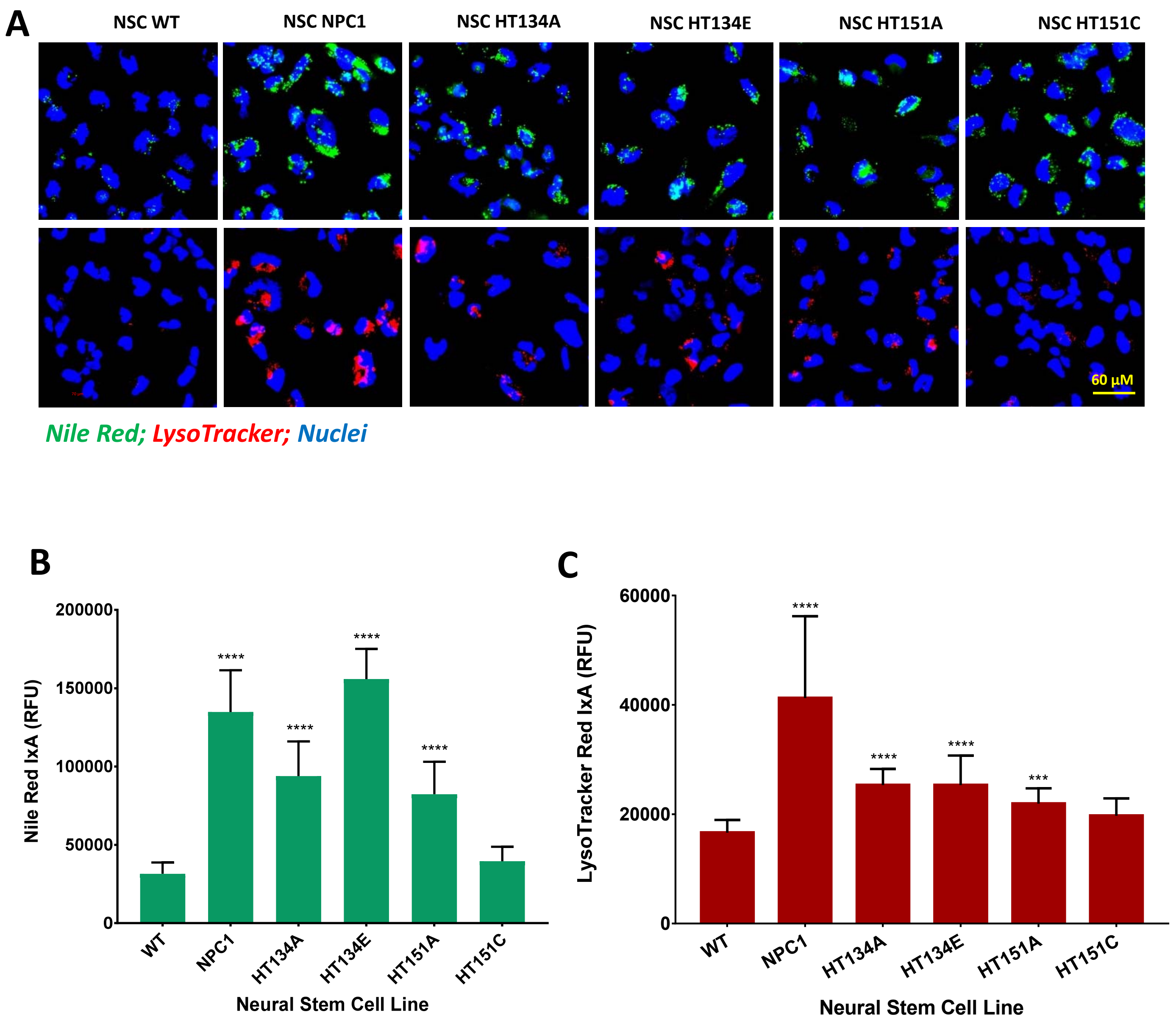

Figure 3. Tay-Sachs disease NSCs express increased lipid accumulation and lysosomal size compared to WT NSCs. A) Images of increased intensity of Nile red and LysoTracker Red staining in TSD NSC compared to WT NSCs after 24hr addition of 10% FBS. The yellow/gold fluorescence of Nile Red excites and emits at 450-500nm and 528nm, respectively. LysoTracker Red excite/emit at 577/590 nm. B) Intensity of Nile Red staining in Tay-Sacs disease NSCs after addition of FBS treatment (n=48; SD; **** $p<0.0001$ compared to WT). Values were calculated via intensity multiplied by the area of the Nile Red staining. C) Intensity of LysoTracker Red staining in Tay-Sacs disease NSCs after addition of FBS treatment (n=48; SD; **** $p<0.0001$ and *** $p<0.001$ compared to WT). Values were calculated via intensity multiplied by the area of the LysoTracker Red staining. NSC NPC1, neural stem cells derived from Niemann-Pick disease, type C (NPC) patient iPS cells, serving as positive control for Nile Red staining and LysoTracker staining. IxA, integrated cell intensity.

Supplemental Fig. 4

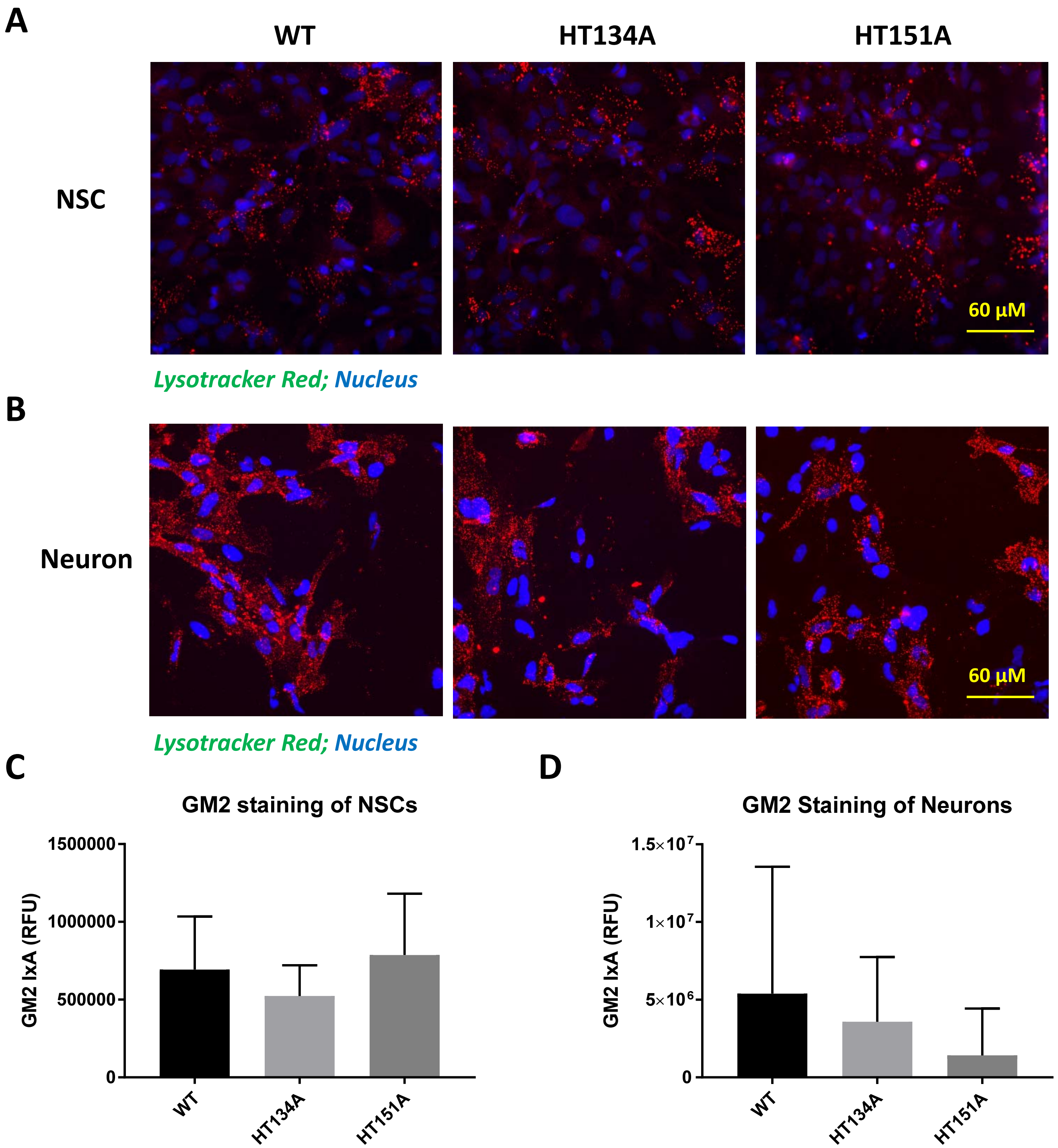

Supplemental Figure 4. Immunofluorescence staining of GM2 in TSD patient NSCs and neurons. A) Immunofluorescence staining of GM2 in TSD NSCs. B) Immunofluorescence staining of GM2 in TSD neurons. C) and D) Quantification data of GM2 immunofluorescence intensity in TSD NSCs and neurons. No significant difference was observed in both TSD NSCs and neurons compared to the WT control NSCs and neurons.

Supplemental Fig. 5

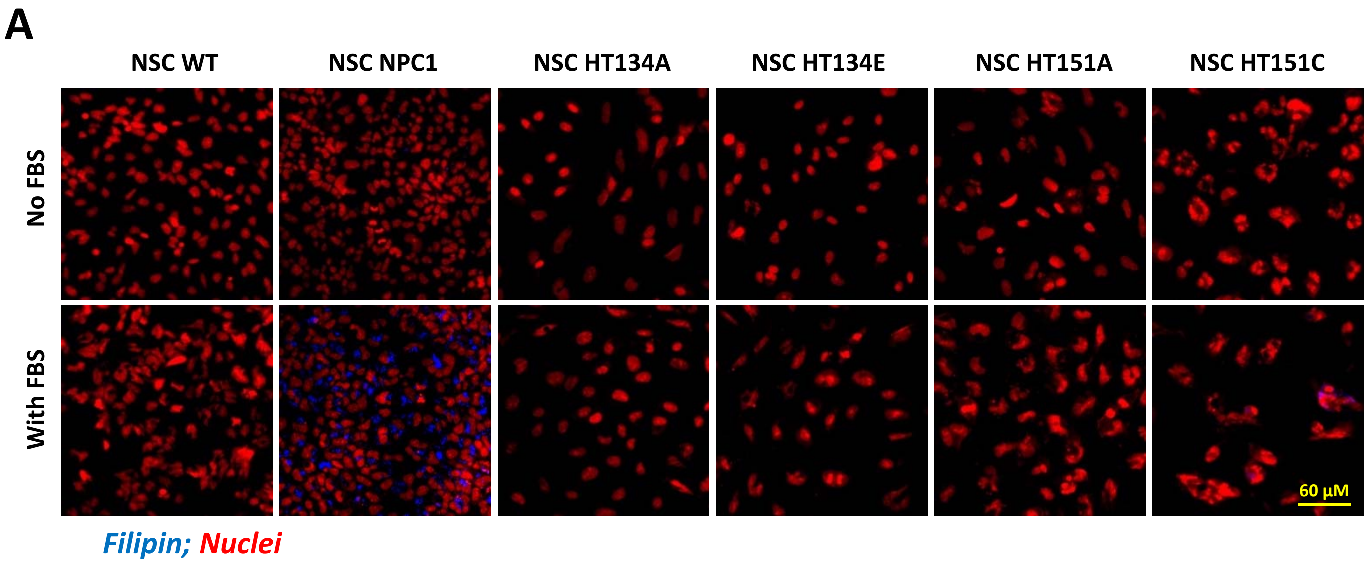

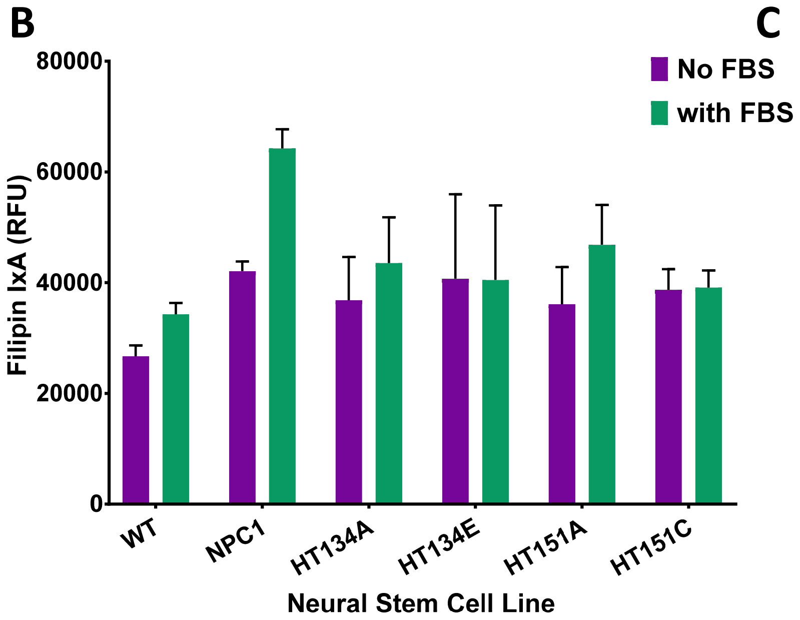

C

NSC Line	Percent Increase in RFU After Addition of FBS
	Integrated Cell Intensity (IxA)
WT (5659)	28.46%
NPC1 (3123)	52.72%
HT134A	18.22%
HT134E	-0.57%
HT151A	29.79%
HT151C	1.03%

Supplemental Figure 5. No significant increase of unesterified cholesterol was detected in TSD NSCs compared to WT NSCs. A) Images of Filipin staining of WT, NPC1, and TSD NSCs before and after 24 hour incubation with 10% FBS. B) Integrated Cell Intensity x Area of Filipin staining of WT, NPC1, and TSD NSCs before and after 24hr incubation with 10% FBS (n=40; mean +/- the standard deviation). C) Percent increase of Filipin staining in WT, NPC1, and TSD NSCs after 24hr addition of 10% FBS. NSC NPC1, neural stem cells derived from Niemann-Pick disease, type C (NPC) patient iPS cells, serving as positive control for Filipin staining. IxA, integrated cell intensity.
